# Ethanol reduces the minimum alveolar concentration of sevoflurane in rats

**DOI:** 10.1038/s41598-021-04364-8

**Published:** 2022-01-07

**Authors:** Johannes Müller, Walter Plöchl, Paul Mühlbacher, Alexandra Graf, Anne-Margarethe Kramer, Bruno Karl Podesser, Thomas Stimpfl, Thomas Hamp

**Affiliations:** 1 Department of Anaesthesia, Intensive Care and Pain Medicine, Division of General Anaesthesia and Intensive Care Medicine, Medical University of Vienna, Vienna, Austria; 2 Institute for Medical Statistics, Center for Medical Statistics, Informatics and Intelligent Systems, Medical University of Vienna, Vienna, Austria; 3 Center for Biomedical Research, Medical University of Vienna, Vienna, Austria; 4 Department of Laboratory Medicine, Medical University of Vienna, Vienna, Austria

**Keywords:** Translational research, Experimental models of disease, Risk factors, Preclinical research

## Abstract

A high number of trauma patients are under the influence of alcohol. Since many of them need immediate surgical procedures, it is imperative to be aware of the interaction of alcohol with general anesthesia. To counter challenges that arise from clinical studies, we designed an animal experiment in which 48 adult Wistar rats either received 1 g · kg^−1^ ethanol, 2 g · kg^−1^ ethanol or placebo via intraperitoneal application. Subsequently, they were anesthetized with an individual concentration of sevoflurane. The minimum alveolar concentration (MAC) of the different groups was assessed using Dixon’s up-and-down design and isotonic regression methods. The bootstrap estimate of the MAC of sevoflurane in the placebo group was 2.24 vol% (95% CI 1.97–2.94 vol%). In the low dose ethanol group, the bootstrap estimate was 1.65 vol% (95% CI 1.40–1.98 vol%), and in the high dose ethanol group, it was 1.08 vol% (95% CI 0.73–1.42 vol%). We therefore report that intraperitoneal application of 1 g · kg^−1^ or 2 g · kg^−1^ ethanol both resulted in a significant reduction of the MAC of sevoflurane in adult Wistar rats: by 26.3% and 51.8% respectively as compared to placebo.

## Introduction

Alcohol is a global contributor to numerous diseases and is associated with a vast number of trauma-related injuries^1–3^. It shows dose dependent stimulative or sedative effects but when consumed in higher quantities central nervous system depressive effects become more pronounced^4^.

Anesthesiologists, emergency doctors, surgeons, and other medical personnel face enormous challenges when confronted with patients who need to undergo surgical procedures but are under the influence of alcohol^5,6^. Each case has to be weighted individually with no definitive guidelines on how to adapt various anesthetic and analgesic agents. Furthermore, in many cases it is not possible to differentiate between chronic alcoholism and acute intoxication^7^.

Inhalational volatile anesthetics play a central role in anesthetic practice. The measurement of end-tidal anesthetic concentration allows titration of the volatile anesthetic and guidance of anesthetic depth. For volatile anesthetics, the standard measure of potency is the minimum alveolar concentration (MAC). It is defined as the concentration at one atmosphere of pressure that prevents gross purposeful movement in response to a surgical incision in 50% of patients^8^. Various drugs such as opioids, ketamine, lidocaine or propofol are known to influence the MAC of volatile anesthetic agents^9–12^. However, experimental and clinical studies researching the effect of ethanol on inhalational anesthetics are scarce and date back to the 1980s. They also mainly focused on chronic effects of alcohol^8,13,14^. To date, we found no experimental or clinical studies investigating the effect of acutely administered ethanol on the anesthetic or sedative effects of sevoflurane, which is one of the most commonly used anesthetic agents^15^.

Controlled clinical studies of the effects of alcohol on sevoflurane requirements in humans are complicated by the fact that different drinking habits such as the frequency or the amount of routinely consumed alcohol are known to influence the effect of acutely administered ethanol. In contrast, trials in patients undergoing emergent surgery under the influence of alcohol lack comparability due to the different degrees of intoxication. To counter these challenges, we used a rat model to assess the effect of two different doses of intraperitoneally applied ethanol on the MAC of sevoflurane.

## Methods

The experiment was approved by the ethics committee for laboratory animal research of the Medical University of Vienna, Vienna, Austria on the 24th of June 2020. Approval of all study procedures was provided by the Austrian Ministry of Education, Science and Research on the 7th of August 2020 (protocol number BMBWF 2020-0.488.784). All experiments were performed according to institutional and federal guidelines and regulations (ICH-GCP, Tierversuchsgesetz 2012).

The study was conducted in October and November 2020 at the Center for Biomedical Research at the Medical University of Vienna, Austria. The study and this manuscript follow the essential and recommended ARRIVE-guidelines 2.0 for animal research^16^. The sample size calculation and statistical planning were performed before the protocol was submitted to the ethics committee.

To determine the effects of alcohol on the MAC of sevoflurane we used an experimental design based on our previously published work^17^. The MAC of sevoflurane was estimated according to the Dixon up-and-down method^18^. Testing of the animals was performed inside an airtight chamber that was flooded with a specific concentration of sevoflurane. A standardized stimulus was applied to the distal third of the rat’s tail as is the common method for determining the MAC in rodents^8^.

### Study animals and housing

We used 24 male and 24 female Wistar rats (Charles River Laboratories, Research Models and Services, Germany GmbH, Sulzfeld, Germany) between the ages of 77 and 95 days to conduct the experiment. Upon arrival, the animals were separated by sex and housed in cages of four (body weight < 400 g) or three (body weight ≥ 400 g). They could acclimate at the facility for 14 days before the start of the experiment. All animals had unrestricted access to autoclaved feed and water. Natural light conditions (light and dark cycles of 12 h each), 22 °C room temperature and relative humidity between 45 and 65% were maintained. At the time of the experiment, the females weighed 278 (17) g (mean (standard deviation)), the males 469 (37) g.

### Testing equipment

We used an airtight transparent chamber (15 cm wide, 20 cm long, 9 cm high) that was flooded with an oxygen/sevoflurane mixture to determine the pain response of each individual rat. The inflow line was connected to a sevoflurane vaporizer (Dräger Vapor® 2000, Dräger Austria GmbH, Vienna, Austria), which was connected to an oxygen-outlet with a constant flow of 3 L · min^−1^.

The outflow line was placed diagonally on the other side of the chamber and led to the in-house gas extraction system. The sevoflurane concentration was measured in the outflow line via a Dräger Primus® (Dräger Austria GmbH, Vienna, Austria) anesthesia device.

The room temperature was held constant at 22 °C. An infrared heating lamp (Manual Mobile Infant Warmer, Fisher & Paykel Healthcare, Auckland, New Zealand) was placed above the testing chamber to prevent hypothermia in the animals. Additionally, a heating blanket provided additional warmth during the experiment. The blanket’s temperature was controlled by a rectal thermometer (Homeothermic Blanket Control Unit, Harvard Apparatus, Holliston, Massachusetts, USA) to ensure normothermia 37.2 (0.3) °C.

### Randomization and blinding

Based on Microsoft Excel’s (Microsoft 365, Microsoft Corporation, Redmond, Washington, USA) rand ()-function, we created a consecutively numbered list of animals before the start of the experiment. The list contained each the rat’s identification number and the testing group (A, B or C). The groups were assigned at random assuring that each group consisted of 8 males and 8 females. For the experiments, the animals were selected arbitrarily by an animal caretaker before they were brought to the testing facility.

Prior to the experiment, a study nurse prepared the study medication. Three different solutions were created and letter-encoded with only the study nurse knowing the composition. It was therefore ensured that the investigators did not know whether the animals had received ethanol or placebo solutions.

Two investigators conducted the study. Investigator one handled the animals, enforced compliance with the protocol, marked the animals and removed the marking on the animal before testing. Investigator two, who was blinded to the animals’ groups, set, and monitored the vaporizer, applied the standardized painful stimulus, and determined the response to the stimulus.

### Study medication

The experiments were conducted one-by-one. For the application of the study medication each animal was placed in the testing chamber individually. Subsequently, the chamber was flooded with 3.5 vol% sevoflurane. After five minutes, the animal was taken out of the box, loss of righting reflex was ensured, and the study medication was administered intraperitoneally.

The animals received either 1 g · kg^−1^ ethanol dissolved in 0.9% saline, 2 g · kg^−1^ ethanol dissolved in 0.9% saline or 0.9% saline without ethanol in the corresponding volume to their body weight (10 ml · kg^−1^).

The animal’s identification number and a letter indicating the blinded group were marked on the base of the animal’s tail. Subsequently, the rat was placed in an empty cage and brought to a separate room for a waiting period of one hour.

### Determination of the MAC

After the waiting period, the rat was put back inside the testing chamber, which was immediately flooded with a mixture of oxygen and sevoflurane. When the animal was unconscious, the box was shortly opened to insert a rectal thermometer and to pass the tail through a therefore intended opening in the chamber. Subsequently, it was sealed to prevent leakage of the sevoflurane/oxygen mixture.

The first rat of each group received 2.6 vol% sevoflurane. After 15 min, a standardized painful stimulus was applied to the distal third of the tail^8^. If the animal showed gross purposeful movement in response to the stimulus, the next animal of the same group received a sevoflurane concentration that was increased by 0.2 percentage points. If there was no movement, the next animal’s sevoflurane concentration was decreased by 0.2 percentage points.

**Figure 1:**
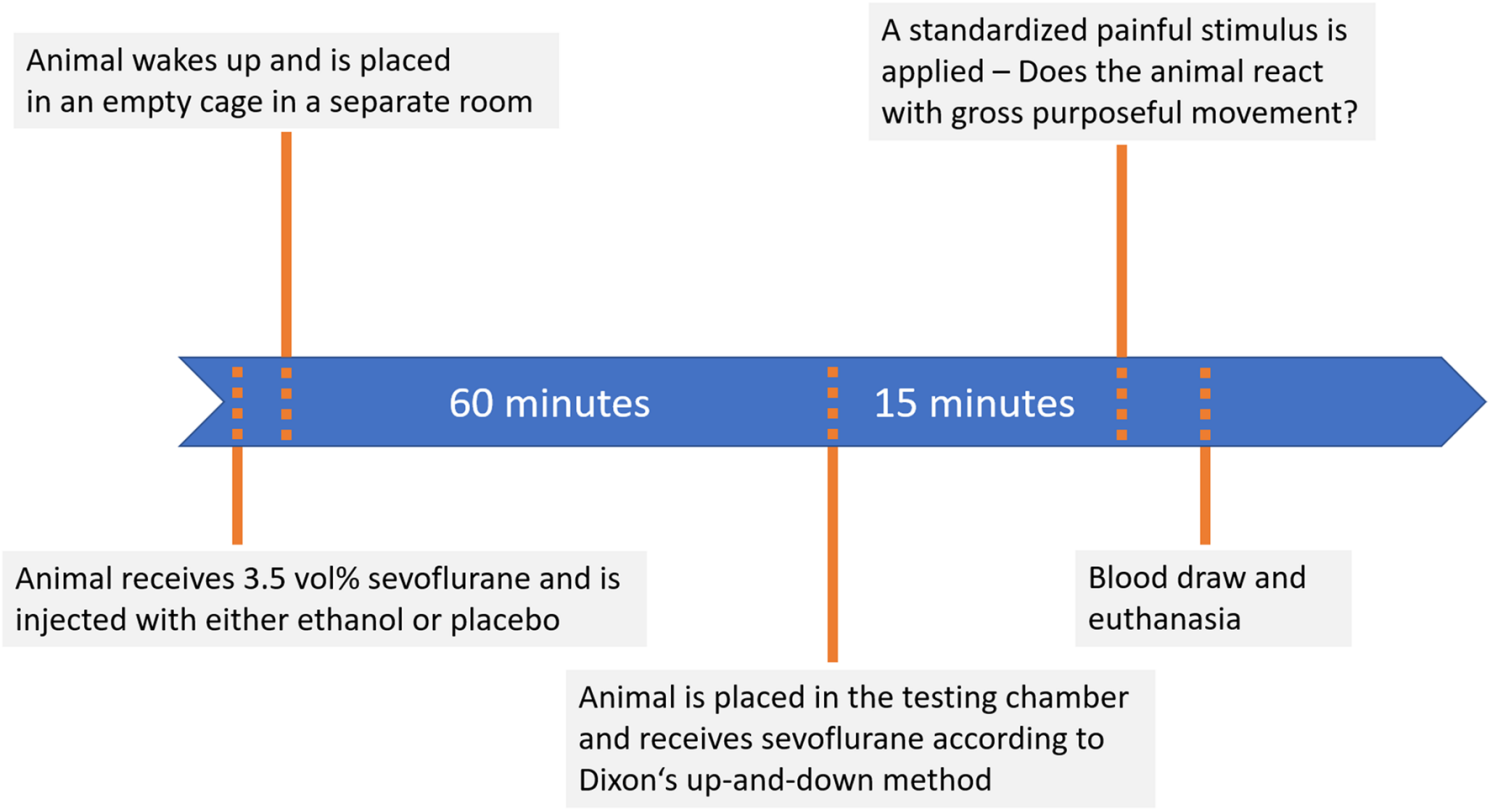
Timeline graphic showing the timing of major events such as intraperitoneal injection and determination of the response to the standardized stimulus.

For the stimulus, we used a surgical clamp (25 cm arterial clamp KLS Martin, Tuttlingen, Germany) that provided 5 locking positions. For all animals, we locked the clamp in the third position, as it provides enough force to trigger the desired stimulus but not so much as to risk amputation of the tail. The clamp stayed locked for either one minute or was removed immediately if the animals showed movement (Fig. 1).

### Serum ethanol concentrations

After the determination of the pain response the animal was placed in front of a breathing tube and received 4 vol% sevoflurane. Five milliliters of blood was drawn via cardiac puncture followed by an intracardial bolus of 300 mg pentobarbital for euthanasia. The blood was centrifuged, and the serum was immediately frozen at -20 °C.

Subsequently, the ethanol concentrations in serum were determined by the enzymatic assay cobas® Ethanol Gen.2 (Roche Diagnostics GmbH, Mannheim, Germany).

### Sample size calculation

Simulations were performed to estimate the power. For the simulation we assumed a dose response-curve with a MAC of µ = 2.8 and standard deviation σ = 0.18 for the Placebo group using the following formula^19^:

$P(response)=\Phi (\frac{dose- \mu }{\sigma })$, where Φ is the cumulative distribution function of the standard normal distribution. In our case, response means that there is no reaction to stimulation. The assumptions on the placebo group were based on a previous experiment in rats^17^.

Since a reduction in the sevoflurane dose of 15% in the MAC between groups was considered to be clinically relevant, the dose response curve for the ethanol groups was assumed with µ = 2.38. The standard deviation was set to σ = 0.18. Dose levels were fixed from 1 to 4 by steps of 0.2. The starting dose was set to 2.6 for all groups to ensure blinding. In each simulation step, the MAC was estimated using isotonic regression and 97.5% confidence intervals (CIs).

Under the given assumptions, simulations showed a power of 80% for a sample size of 16 rats per group.

### Statistical analysis

The MAC values of the three groups (high-dose ethanol, low-dose ethanol and placebo) were estimated using isotonic regression methods^19,20^. The corresponding bootstrap estimator and the CIs were calculated separately for the groups as well as for the difference in the MAC values between the two groups and placebo using 5000 bootstrap samples. For the difference, 97.5% CIs were calculated due to the two comparisons to placebo.

The analyses were performed using R v.3.3.3 (R: A Language and Environment for Statistical Computing, R Core Team, R Foundation for Statistical Computing, Vienna, Austria 2014).

## Results

**Figure 2:**
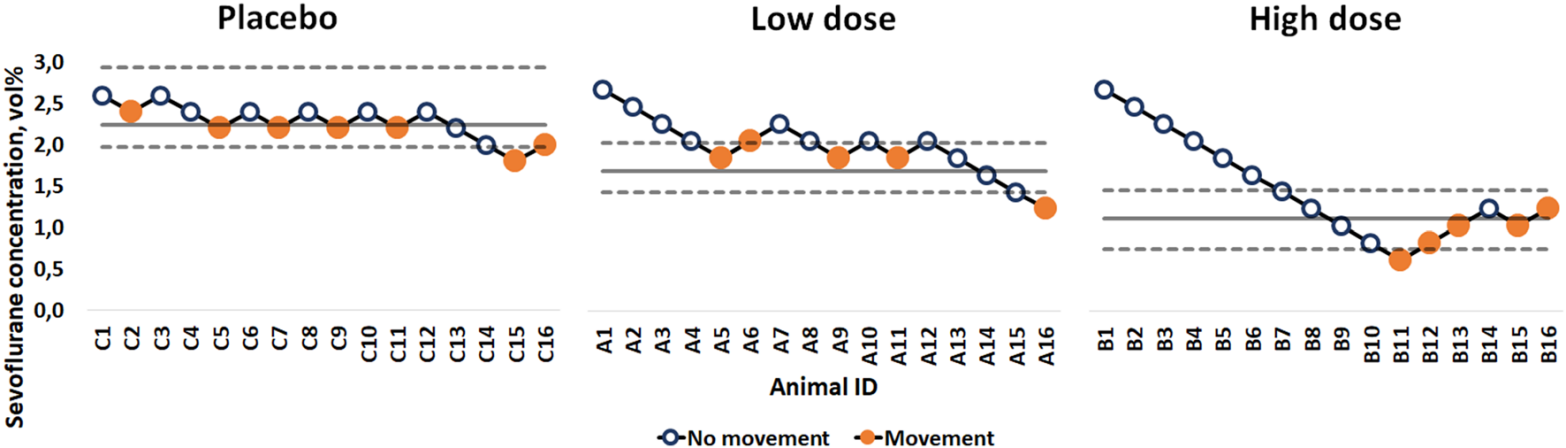
Bootstrap estimates for the MAC (solid horizontal lines) and the confidence intervals (dashed horizontal lines). Gross purposeful movements are shown as full dots, and no movements are shown as circles. X-axis: Animal ID, Y-axis: sevoflurane concentration (vol%).

The bootstrap estimate of the MAC of sevoflurane in the placebo group was 2.24 vol% (95% CI 1.97–2.94 vol%). In the low dose ethanol group, the bootstrap estimate was 1.65 vol% (95% CI 1.40–1.98 vol%) while in the high dose ethanol group it was 1.08 vol% (95% CI 0.73–1.42 vol%) (Fig. 2).

The CIs of the difference in MAC values between the two dose groups and placebo did not contain 0, indicating a significant difference in the MAC of sevoflurane between the high dose ethanol and the placebo group as well as the low dose ethanol and placebo group (Table 1).

**Table 1:** Sample estimates and bootstrap estimates of the MAC and the corresponding bootstrap confidence intervals.

Group	Sample estimate	Bootstrap estimate	Bootstrap lower CI	Bootstrap upper CI
Low-dose ethanol	1.70	1.65	1.40	1.98
High-dose ethanol	1.03	1.08	0.73	1.42
Placebo	2.24	2.24	1.97	2.40
Placebo—low dose	0.54	0.59	0.07	0.93
Placebo—high dose	1.21	1.17	0.71	1.58

**Figure 3:**
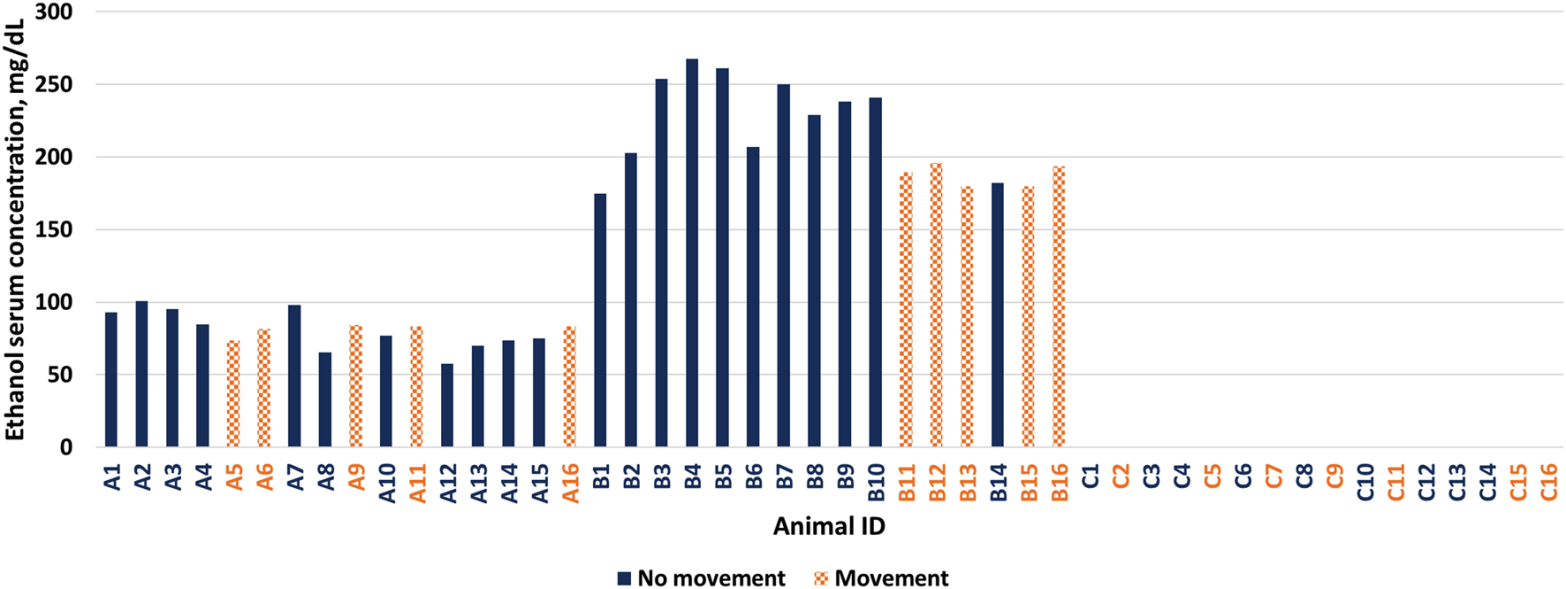
Serum ethanol concentrations per animal. A solid bar represents a negative reaction, a dotted bar a positive reaction. X-axis: Animal ID, Y-axis: Serum ethanol concentrations (mg · dL^−1^).

The mean serum ethanol concentrations were 80.97 (11.52) mg · dL^−1^ in the low dose ethanol group and 215.31 (31.51) mg · dL^−1^ in the high dose ethanol group. In the placebo group, no ethanol was detected in the serum (Fig. 3).

## Discussion

In this observer blinded animal experiment we report a statistically significant reduction in the MAC of sevoflurane by ethanol. In rats, the intraperitoneal application of 1 g · kg^−1^ ethanol decreased the MAC of sevoflurane by 26.3% and the intraperitoneal application of 2 g · kg^−1^ ethanol decreased the MAC by 51.8% compared to placebo.

We found the MAC in our control group to be 2.24 vol%, which is lower than we had expected. Various authors reported the MAC of sevoflurane in Wistar rats to be between 2.29 vol% and 2.8 vol%, which was our reason for starting each group at 2.6 vol% sevoflurane^17,21,22^. However, there are many factors that influence the MAC such as age or their individual levels of stress hormones^8,22^. Additionally, Gong and colleagues found that different strains of animals present with different anesthetic requirements^21^. This finding highlights the necessity of control groups in experimental studies and shows that historical controls are insufficient when designing experiments to determine the MAC of volatile anesthetics.

Alcohol is a nearly globally consumed drug that is widely socially accepted. Given that up to 50% of trauma-related injuries occur under the influence of alcohol, it is critical to consider its interaction with general anesthetics^1–3^. The effects of alcohol are dose dependent. Low doses may result in anxiolytic and euphoric effects, while high doses lead to sedation and central nervous system depression and may even result in coma^4^.

Ethanol binds and modulates various receptors, including gamma-aminobutyric acid (GABA)^23^, N-methyl-D-aspartate (NMDA)^24^, nicotinic acetylcholine (nAChR)^25^, serotonin (5-HT)^26^ and glycine (Gly)^27^. There is evidence that sevoflurane also targets these systems, which might explain similar pharmacological effects^28–31^.

Acute intraperitoneal administration of ethanol reduces the MAC of isoflurane in mice, as shown by Johnstone and colleagues^13^. Wolfson and Freed found reduced halothane requirements in rats that were given ethanol intraperitoneally^32^. Our data appear to be consistent with the finding that acutely administered ethanol shows the ability to reduce the MAC of various volatile anesthetic agents.

One common challenge of animal models lies in the fact that it is difficult to compare dosages between animals and humans. Bodyweight adjusted applications across different species may result in pronounced or reduced effects which might be explained by numerous factors, such as differences in body surface to volume ratio, metabolism, and perfusion. Various studies have tried to determine behavioral and social effects evoked by different bodyweight adjusted dosages of ethanol in rats. These behavioral changes can be compared to those found in human studies.

Varlinskaya and colleagues reported social facilitation in low doses of ethanol (0.25–0.75 g · kg^−1^) and social inhibition in higher doses (3–4 g · kg^−1^) in rats after intragastric administration^33^. Miranda-Morales and colleagues found anxiolytic properties of low doses of intraperitoneally administered ethanol (0.5 g · kg^−1^) in infant rats^34^. In humans, similar properties are present at comparable dosages. Conventional wisdom and multiple textbooks describe social facilitation, euphoria and talkativeness as effects of low doses of alcohol in humans while higher doses result in apathy and may even induce unconsciousness and respiratory arrest^35,36^. However, exact dosage comparison between rats and humans is imperfect because bodyweight-adjusted dose–response trials are limited by factors like the individuals’ drinking habits or genetic predispositions. Although it was our aim to choose an experimental design, which simulated the situation in humans as well as possible, we acknowledge the limitation that our model lacks the ability to compare dosages 1:1 between rats and humans.

We aimed to test for serum ethanol concentrations that are realistic for trauma patients and therefore clinically relevant. To establish these concentrations, we compared different legal driving limits across the world. Blood ethanol concentrations are often measured as the mass of alcohol per volume of blood or mass of alcohol per mass of blood, depending on the country and the system of units. In many European countries, the limit to legally drive a passenger car is 0.5 g · L^−1^. Factoring the density of blood serum (1.026 g · mL^−1^) and the distribution coefficient of serum to whole blood (1.2) this legal limit converts to 61,56 mg · dL^−1^ ethanol in blood serum^37^. The ethanol concentrations we measured in serum therefore seem to be highly clinically relevant at approximately 1.3 and 3.5 times this converted limit. They are also in line with findings by Rivara and colleagues, who reported mean blood alcohol concentrations of trauma patients to be 1.86 g · L^−1^.^38^ A recent study conducted by Riuttanen and colleagues. found that intoxicated trauma patients in Finland present with a mean blood alcohol concentration of 1.9 g · L^−1^, which is just slightly above our high-dose ethanol group (1.75 g · L^−1^)^1^.

Methodologically, we need to acknowledge one limitation regarding Dixon’s up-and-down method. The first ten rats in the high-dose ethanol group showed no reaction to the stimulus until gross purposeful movement was observed. Since it is primarily the data points where a change from no movement to movement or vice-versa is used to estimate the MAC, confidence intervals in this group are higher. However, given the strong effect, the reduction in the MAC is statistically significant.

One major strength of Dixon’s method arises from the fact that fewer data points are needed to estimate the MAC compared to other methods^18,19^. In our experiment, the animals that showed no movement were, in general, associated with higher ethanol serum concentrations. Given that there is some variability of serum concentrations to be expected and that there appears to be a dose-dependent effect of ethanol, more data points may have resulted in an even more confident result.

A possible limitation of our experimental design might be found in the fact that one of the two investigators was not blinded to the group the animals belonged to (A, B or C but not whether they had received ethanol or placebo), which might have introduced bias. We tried to mitigate this circumstance by making sure that the second investigator who set the vaporizer to the determined level, applied the painful stimulus, and determined the response to the stimulus, was not aware of this information.

We used sevoflurane to facilitate intraperitoneal injections in order to reduce stress for the animals and limit potential bias as stress hormones such as catecholamines have been shown to increase the MAC^8^. Our literature search did not produce any studies that suggest that the MAC of sevoflurane or other volatile anesthetics is dependent on previous exposures. We regard this to be unlikely since the MAC is unaffected by the duration of anesthesia^39^. Still, we cannot rule out that such an effect exists, potentially introducing bias to our study design.

We regard our findings to be of clinical importance, especially in multidisciplinary settings such as emergency or trauma medicine. For example, in resuscitation, patients are often in a state of hypovolemic shock^40^. The resulting hemodynamic instability can be exacerbated by unnecessarily deep anesthesia. It is therefore imperative to be aware of the acute effects of alcohol intoxication on anesthetic potencies.

In conclusion, intraperitoneal application of 1 g · kg^−1^ ethanol or 2 g · kg^−1^ ethanol significantly reduced the MAC of sevoflurane in adult Wistar rats compared to placebo. The reduction appears to be dose-dependent and is present in clinically relevant doses. There is reason to believe that this effect is also present in humans. However, to assess more exact quantitative and qualitative data in humans, controlled clinical trials are needed.

## Data Availability

The datasets generated during the current study are available from the corresponding author on reasonable request.
